# Supplementary material for: Integrating the Soil Microbiota and Metabolome Reveals the Mechanism through Which Controlled Release Fertilizer Affects Sugarcane Growth
Source: Int J Mol Sci. 2023 Sep 14;24(18):14086. doi: 10.3390/ijms241814086 (PMC10531416; doi:10.3390/ijms241814086)
Supplement: Supplementary file 1 [file ijms-24-14086-s001.zip › ijms-2585000-supplementary.pdf]

Table S1 Controlled release fertilizer formulas

Name	Content (%)	Proportion (%)
Ammonium nitrate	35	14.63
Ammonium chloride	25.3	7.8
Ammonium sulfate	20.5	6.83
Urea	46	22.13
Calcium, magnesium, phosphorus	18	8.78
Potassium polyphosphate	88	12.5
Potassium chloride	60	16.58
Ferrous Sulfate	100	0.425
Magnesium sulfate	100	0.85
Zinc Sulfate	100	0.165
Borax	100	0.285
Polyglutamic acid	30	0.125
Anti-photolysis agent	100	0.005
Anti-caking powder	100	0.6
Anti-sloughing oil	100	0.375
Thiamethoxam	99	0.1
Insecticide	95	0.1
Organic Fertilizer	80	complement

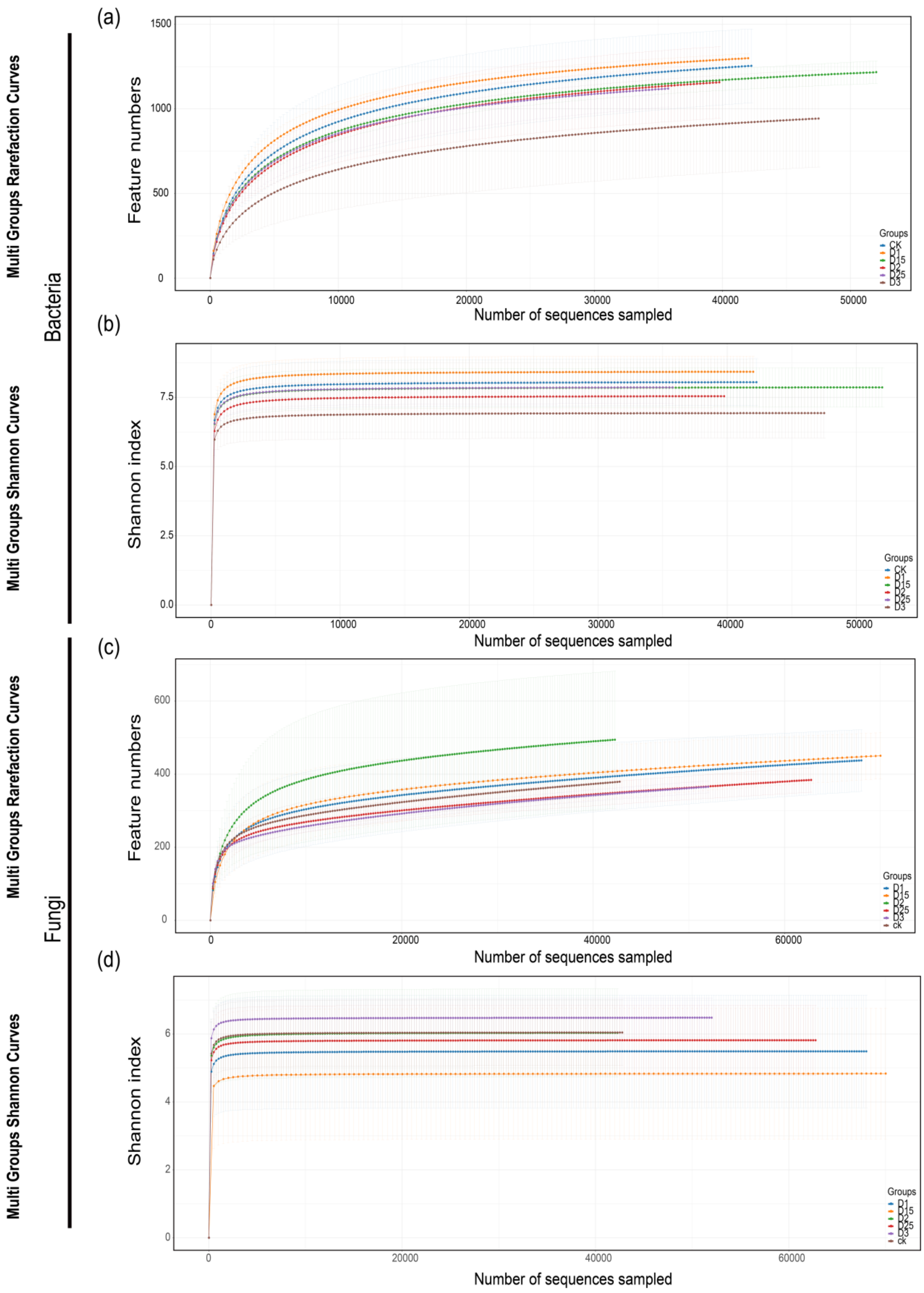

Figure S1 Rarefaction curves for bacteria (a and b) and fungi (c and d).

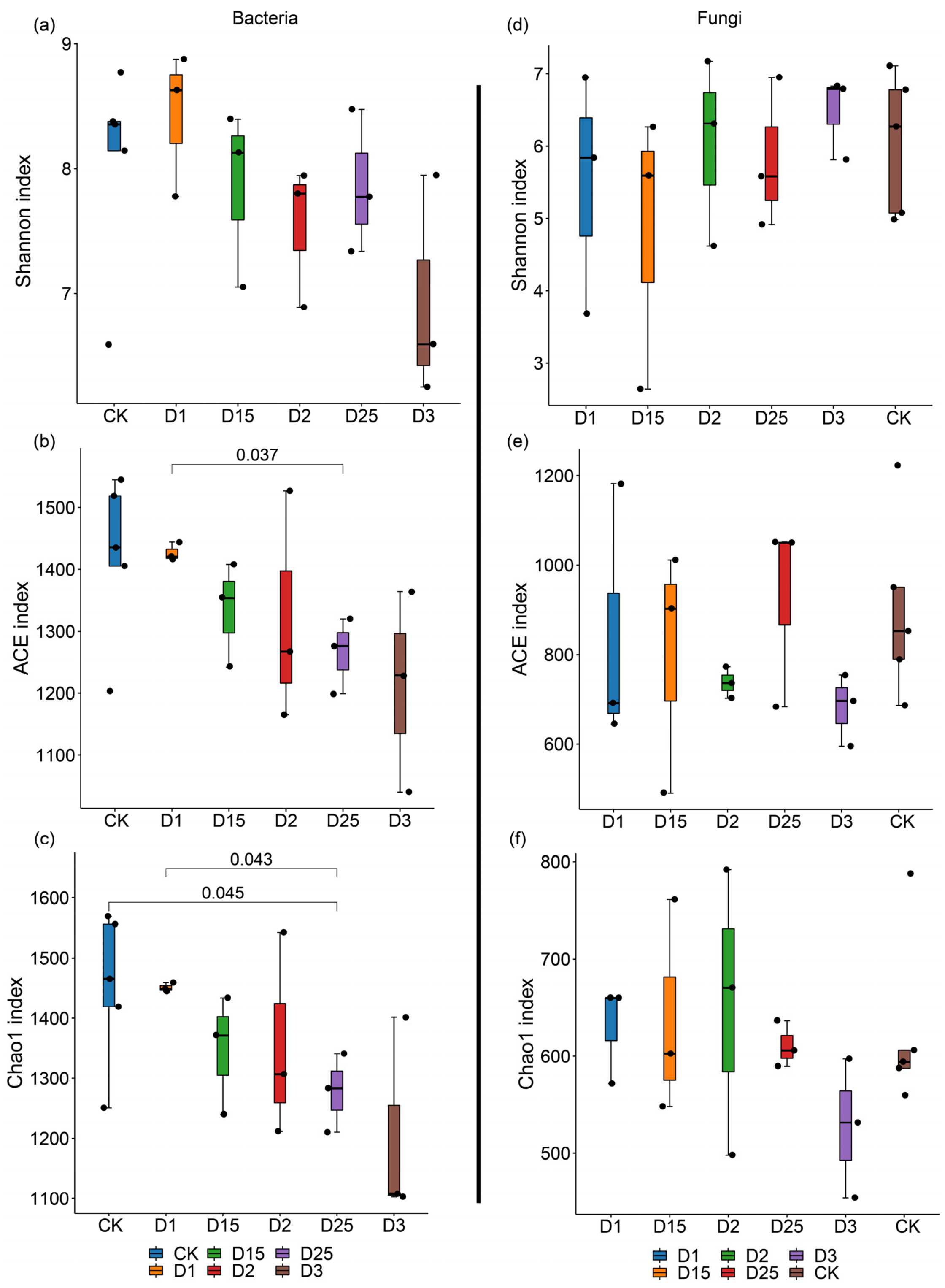

Figure S2 Box line plot of alpha diversity index for microbial communities based on Students t-test test conditions, including Shannon (a and d), ACE(b and e), and Chao1 index (c and f).

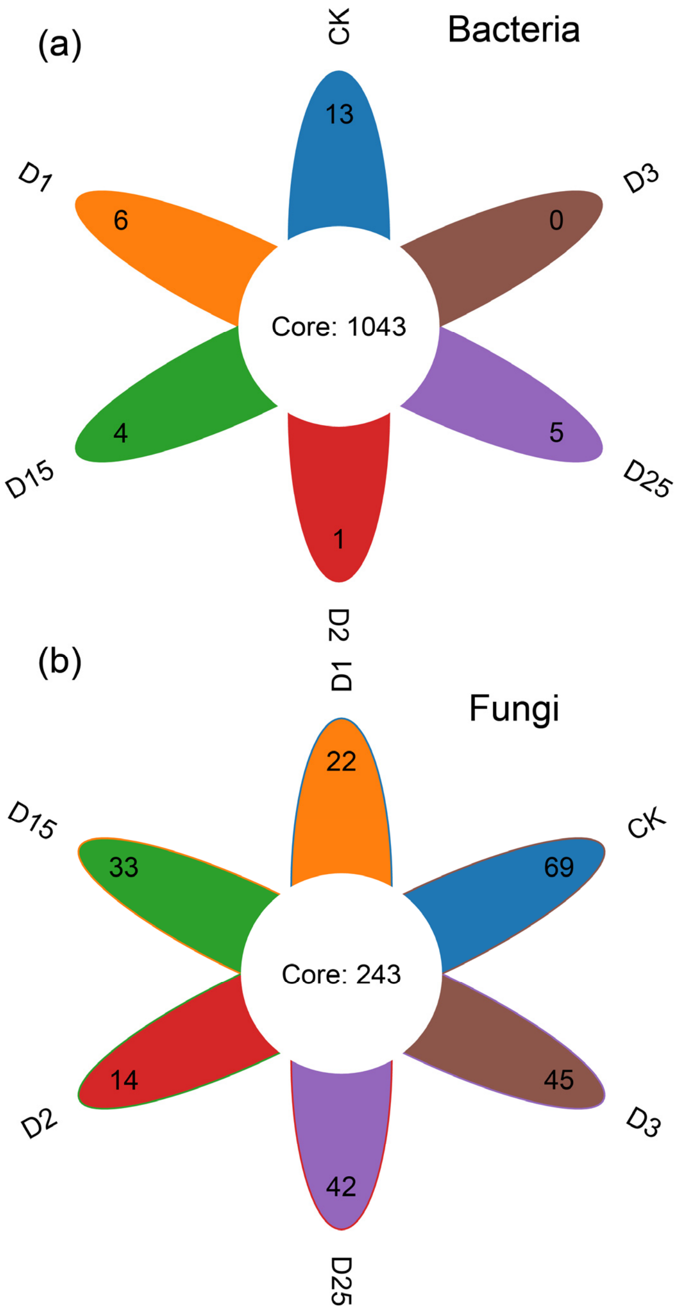

Figure S3 Venn diagram for different treatment conditions based on bacterial (a) and fungal (b) OTU levels.

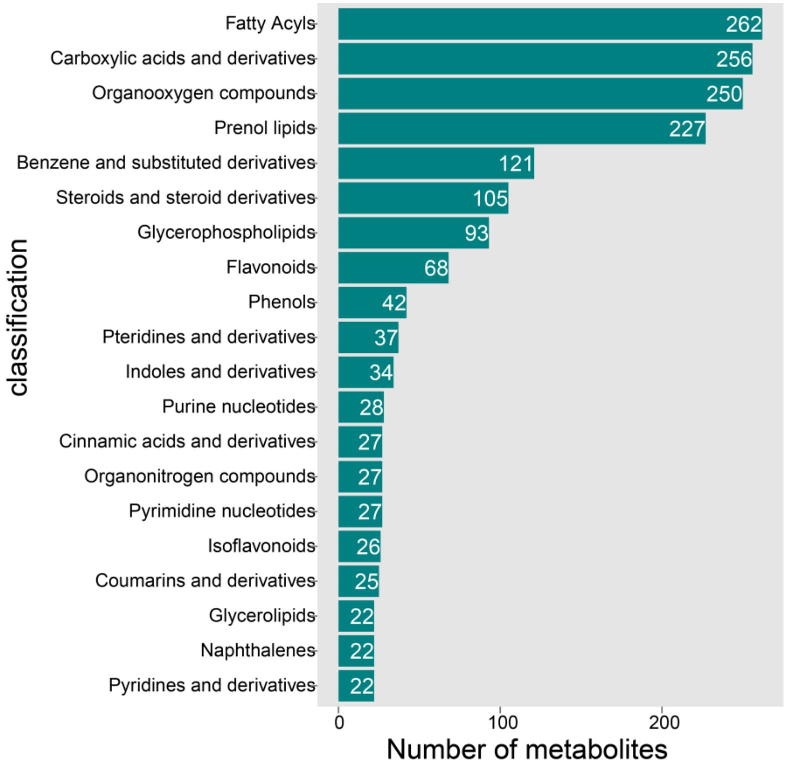

Figure S4 Metabolites under different database annotations. Top 20 metabolite classes based on HMDB (Human Metabolome Database) database annotations.

Table S2 Summary of the number of changes in differential metabolites across comparison groups

Group	DEMs_total	DEMs_up	DEMs_down
D15_vs_D2	96	82	14
D15_vs_D25	24	8	16
D15_vs_D3	56	34	22
D1_vs_D15	27	12	15
D1_vs_D2	45	24	21
D1_vs_D25	33	9	24
D1_vs_D3	57	24	33
D25_vs_D3	4	2	2
D2_vs_D25	37	8	29
D2_vs_D3	51	17	34
CK_vs_D1	80	49	31
CK_vs_D15	71	51	20
CK_vs_D2	102	84	18
CK_vs_D25	19	17	2
CK_vs_D3	21	12	9

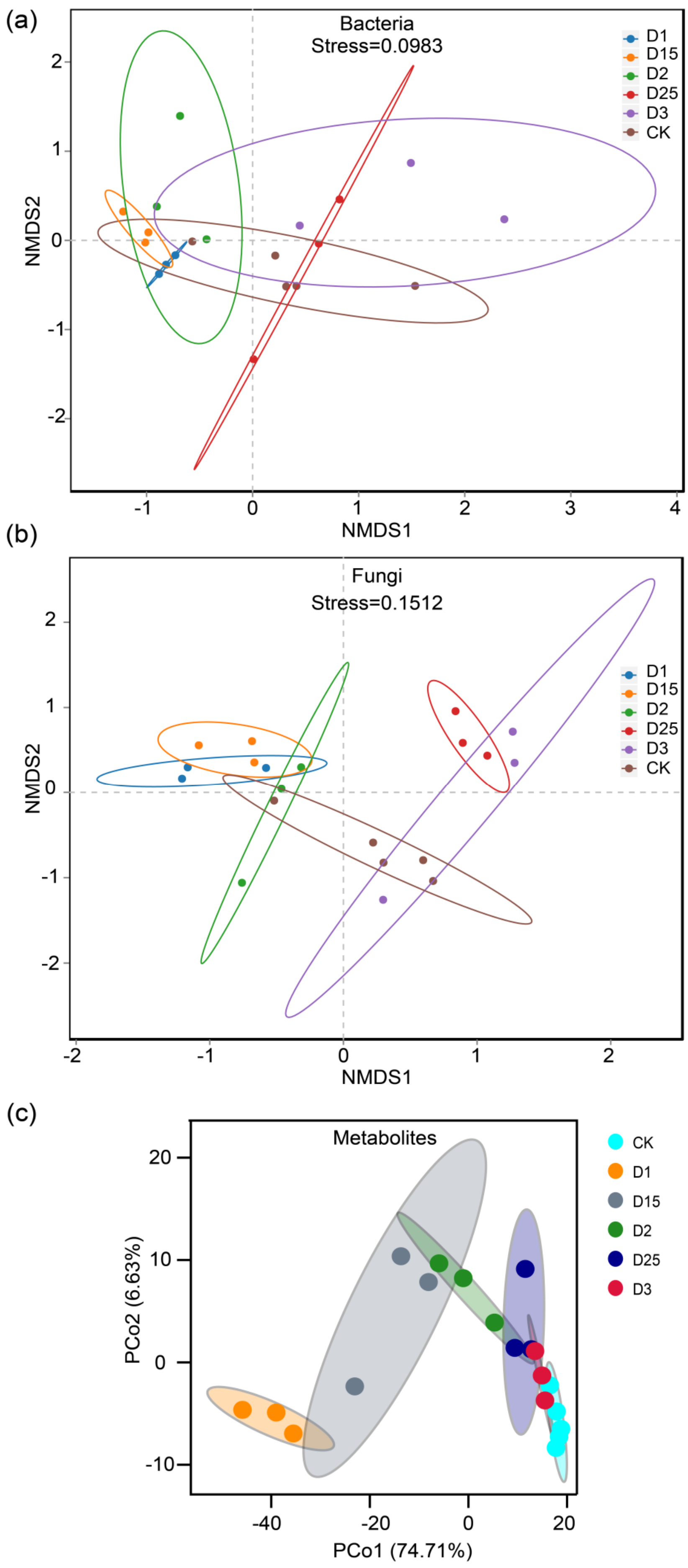
(a)
Bacteria
Stress=0.0983
D1
D15
D2
D25
D3
CK
NMDS2
NMDS1
2
1
0
-1
-2
-1
0
1
2
3
4
(b)
Fungi
Stress=0.1512
D1
D15
D2
D25
D3
CK
NMDS2
NMDS1
2
1
0
-1
-2
-2
-1
0
1
2
(c)
Metabolites
CK
D1
D15
D2
D25
D3
PCo2 (6.63%)
PCo1 (74.71%)
20
10
0
-10
-40
-20
0
20

Figure S5 Bacterial (a) and fungal (b) NMDS analysis based on binary-jaccard distance. PCoA analysis of metabolites based on bray-curtis distance (c). Different colors represent different CRF application rates.

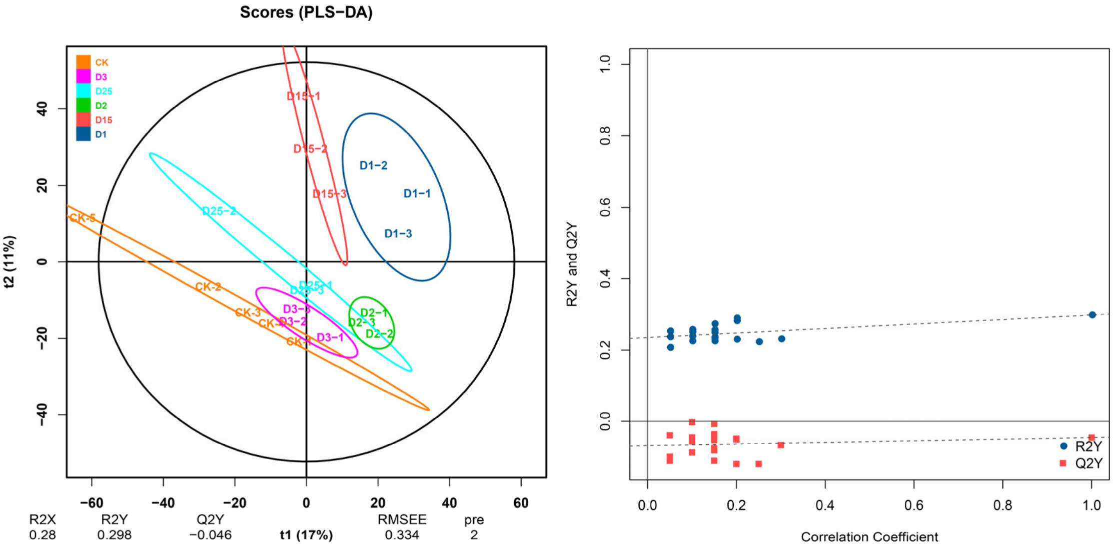

Figure S6 The t2 axis is the orthogonal component (within-group variance component); and the blue and red dots in the cross-validation model are R2Y and Q2Y of the model after replacement, respectively; and the dashed lines are the regression lines of R2Y and Q2Y fits.

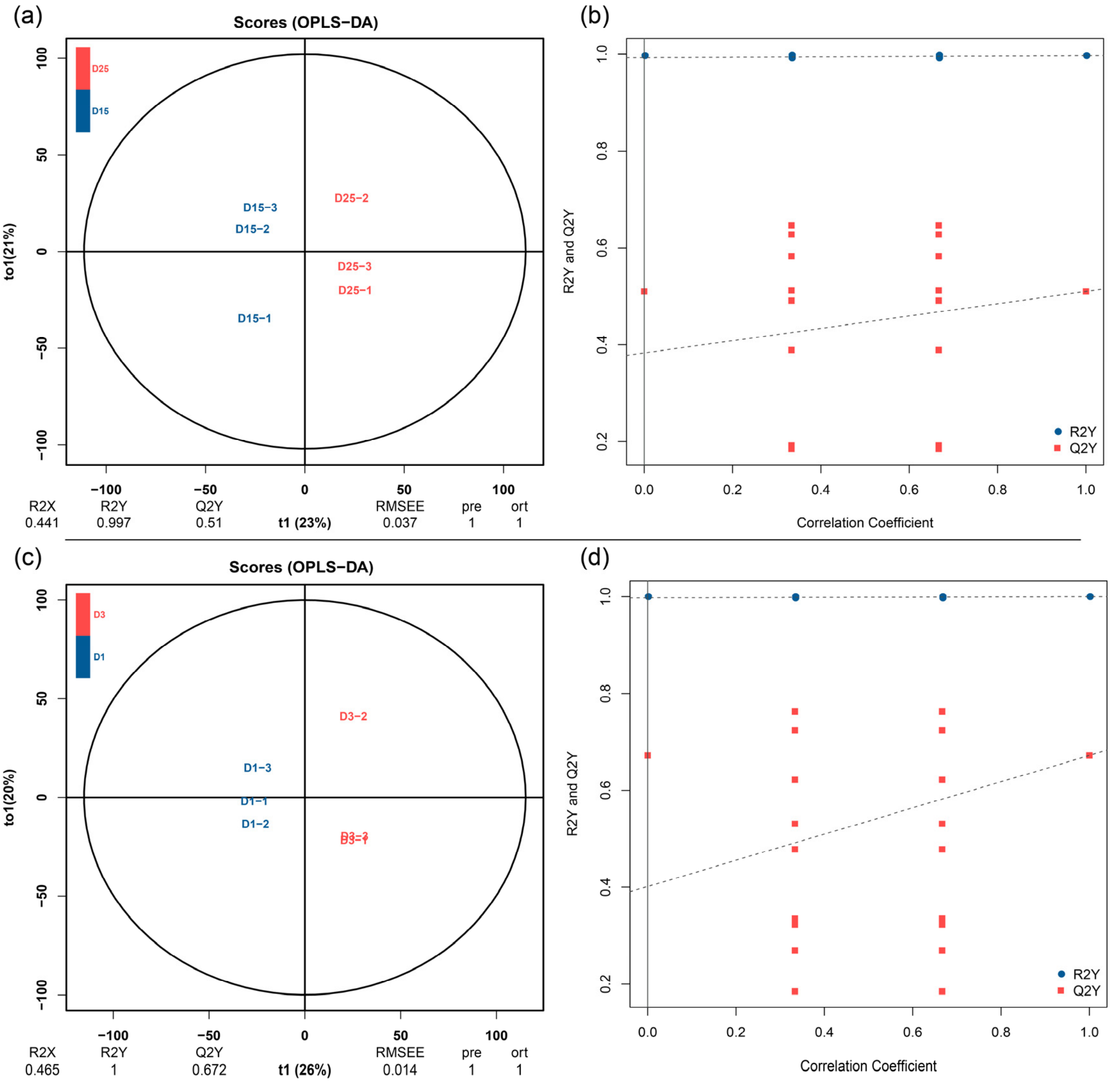

Figure S7 OPLS-DA based on different comparison groups. The x-axis represents the between-group variance component, the y-axis represents the within-group variance component, and the horizontal y-axis percentage represents the component's share of the total variance. D15 vs. D25 (a and b), D1 vs. D3 (c and d).
